# Resonant Andreev Spectroscopy in normal-Metal/thin-Ferromagnet/Superconductor Device: Theory and Application

**DOI:** 10.1038/srep17544

**Published:** 2015-12-02

**Authors:** Francesco Romeo, Filippo Giubileo, Roberta Citro, Antonio Di Bartolomeo, Carmine Attanasio, Carla Cirillo, Albino Polcari, Paola Romano

**Affiliations:** 1 Dipartimento di Fisica “E. R. Caianiello”, Università di Salerno, Fisciano, Italy; 2 CNR-SPIN Salerno, via Giovanni Paolo II 132, Fisciano, Italy; 3 Dipartimento di Scienze e Tecnologie, Università del Sannio, Benevento, Italy

## Abstract

We develop a theoretical model to describe the transport properties of normal-metal/thin-ferromagnet/superconductor device. We perform experimental test of the model using a gold tip on PdNi/Nb bilayer. The resonant proximity effect causes conductance features very sensitive to the local ferromagnetic properties, enabling accurate measurement of polarization and thickness of the ferromagnet by point contact spectroscopy.

Spin polarization (*P*) represents an intrinsic parameter that characterizes a ferromagnet measuring the spin imbalance for the occupied electronic states. Experimentally, *P* can be determined by photoemission spectroscopy^1^ (PS) as well as by spin-dependent spectroscopy on magnetic tunnel junctions^2^ (MTJs), but both methods have important drawbacks: PS has limited energy resolution (few meV) and spatial sensitivity (few Angstrom of the surface) while MTJs need high quality fabrication process to get planar structures with uniform thin insulating barrier and a setup to apply high magnetic fields.

Less than twenty years ago, De Jong and Beenakker^3^ proposed the possibility to measure *P* by means of point contact Andreev reflection (PCAR) spectroscopy exploiting the Andreev reflection (AR) process at the metal(N)/superconductor(S) interface for which an incoming electron with energy less than the superconducting energy gap is retroreflected in the metal as a hole with opposite spin, while a Cooper pair enters the superconductor^4^. If the metal is a ferromagnet (F), the probability for AR is reduced and (transport) polarization can be obtained from the study of the differential conductance spectra, as experimentally reported in 1998 by Soulen *et al*.^5^ and by Upadhyay *et al*.^6^. Later, this technique has been used to characterize several ferromagnetic metals^5^,^6^,^7^,^8^ (Fe, Co, Ni), alloys^5^,^9^ (permalloy Ni_*x*_Fe_1−*x*_), manganites^5^,^10^,^11^ (La_1−*x*_Sr_*x*_MnO_3_), ruthenates^12^,^13^ (SrRuO_3_) and half metals^5^,^14^,^15^ (CrO_2_).

From a theoretical point of view, a simple approach by Strijkers *et al*.^7^ gives a generalization of the Blonder-Tinkham-Klapwijk (BTK) theory^16^ to spin polarized materials by considering the current flowing in a F/S contact as *I* = (1 − *P*) ⋅ *I*_*u*_ + *P* ⋅ *I*_*P*_ with *I*_*P*_ and *I*_*u*_ the fully polarized and fully not-polarized current, respectively. Their model considers a weak (proximized) superconducting layer at the interface and succeeded, in some cases, to fit conductance dips often experimentally observed at energies close to the gap energy. Alternatively, F. Peréz-Willard *et al*.^17^ have considered two spin-dependent transmission coefficients for the majority and minority carriers in the ferromagnet. Both models have been widely applied to extract spin polarization in several PCAR experiments involving ferromagnetic materials. This powerful method probes the bulk polarization of the ferromagnet (used as one of the electrodes) in the F/S contact. In this case, the boundary conditions are determined by the bulk density of states (DOS) in F, and consequently the Andreev reflection probability is reduced by the lack of DOS available for the electron-hole scattering.

The scenario described above is modified when PCAR spectroscopy is used to characterize F/S bilayers where a thin F-layer covers the S-electrode, a configuration of technological interest in the field of magnetic nano-devices. The analysis of such system requires further theoretical investigations, specially in the presence of very thin ferromagnetic layer. Indeed, the use of metallic (non-magnetic) tip as electrode realizes a N-F/S device in which the N-electrode fixes the boundary conditions. In this case, the Andreev probability is not limited by the lack of DOS, as for a bulk ferromagnetic electrode configuration, while it is strongly affected by interference phenomena caused by resonant proximity effect. The resonant proximity originates from the influence of Andreev bound states confined to the F-layer and can be extremely sensitive to the ferromagnetic properties (thickness and polarization). For these reasons, resonance effects can be exploited to implement a Resonant Andreev Spectroscopy technique useful for the characterization of magnetic systems of reduced dimensions.

In this work we develop a theoretical model within the Bogoliubov-de Gennes (BdG) formalism^18^ taking into account the presence of the tip/sample (N/F) and F/S (in the bilayer) interface, which correctly reproduces the double barrier geometry of a Resonant Andreev Spectroscopy setup. The theory demonstrates that N-F/S configuration allows precise estimation of spin polarization as well as of the ferromagnet local thickness. A first evidence of the applicability of the model is provided by analyzing results obtained in PCAR experiment using a gold tip on the ferromagnetic side of a PdNi/Nb bilayer.

## Results

### Theoretical model

Up to now, double barrier problem, relevant in describing a Resonant Andreev Spectroscopy setup, has been studied for diffusive N/X/S systems (with X indicating a normal metal or a ferromagnet) by using Usadel equations^19^,^20^. The semiclassical nature of this approach^21^ requires the existence of a semiclassical solution in the interlayer which is only allowed for a minimum interlayer thickness (i.e. the distance between the two barriers) much larger than the Fermi-wavelength. For smaller thickness, the double barrier transmission coefficients are strongly affected by quantum-mechanical coherence. Thus, for the N-F/S system with reduced thickness of the F-interlayer (~*ξ*_*F*_, where *ξ*_*F*_ is the characteristic length of the superconducting correlation decay in the F-layer), we adopt a Bogoliubov-de Gennes formalism in which the wave function Ψ(*r*), describing an excitation of energy *E* in the tip, in the ferromagnetic layer or in the superconductor, is derived by solving the eigenvalues problem given by (*x* ≠ 0, *d*)


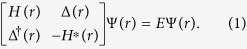


**Figure 1:**
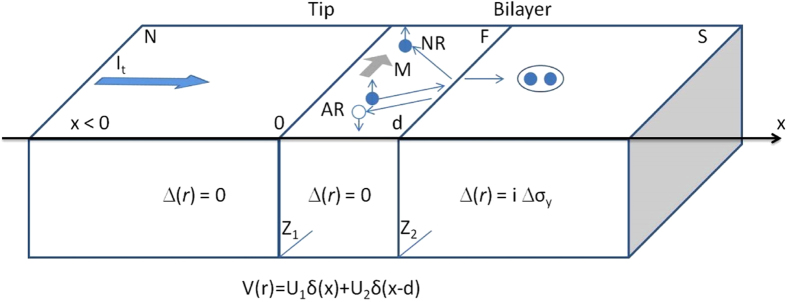
Schematic representation of a Resonant Andreev Spectroscopy setup. Transport current *I*_*t*_ flows from metallic tip through N/F (parameterized by *Z*_1_) and then F/S (*Z*_2_) interfaces, barrier strengths depending on the scattering potential *V*(*r*). Ferromagnetic region (0 < *x* < *d*) has magnetization *M* perpendicular to transport direction. Superconducting pairing potential Δ(*r*), existing in S, causes Andreev reflection (AR), while the normal reflection (NR) at F/S and N/F interface is caused by *V*(*r*).

In Fig. 1 we give a schematic of the model. The picture evidences the three-dimensional character of the bulk normal electrode (tip) which provides the correct boundary condition for the scattering problem of electronic processes generated asymptotically far from the interface. This is the standard procedure applied in the scattering theory and used in the BTK approach^16^: the scattering coefficients are calculated by considering the normal tip as a layer of infinite transverse dimension^22^,^23^,^24^, as detailed hereafter.

The tip region (*x* < 0), does not present superconducting correlations (Δ(*r*) = 0), while 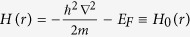 is the tip quasi-particle Hamiltonian. The thin ferromagnetic layer (0 < *x* < *d*) is modeled by adding to *H*_0_(*r*) a Zeeman energy term, 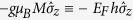, describing a magnetization *M* belonging to the *y* − *z* easy plane orthogonal to the transport direction (*x*-direction). The superconducting region (*x* > *d*) is described by a homogeneous pairing potential 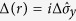. The Fermi velocities mismatch among the different regions and the non-ideality of the interfaces are modeled by using a scattering potential *V*(*r*) = *U*_1_*δ*(*x*) + *U*_2_*δ*(*x* − *d*), *δ*(*x*) being the Dirac delta function. The translational invariance implies the conservation of the linear momentum 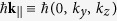 parallel to the interface. Thus, the wave function in each region can be written in the form 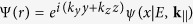 leading to an effective one-dimensional problem for 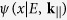, being the energy *E* and 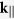 conserved quantum numbers during a scattering event. Once the wave functions 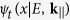, 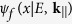 and 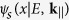 describing, respectively, the tip, the magnetic layer and the superconductor have been expressed in terms of eigenfunctions associated to the eigenvalues problem given in equation (1), the scattering coefficients are determined by imposing the boundary conditions:


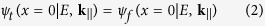



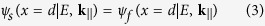



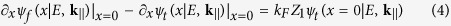



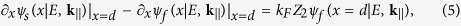


where *k*_*F*_ indicates the Fermi wave vector, while 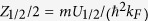 represents the BTK parameter describing the interface properties. The current *I*_*t*_ flowing through the device is expressed via the AR coefficients 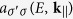 and the normal reflection coefficients 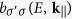 defining the tip wave function 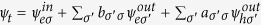. Here *ψ*_*t*_ is decomposed into incoming (in) or outgoing (out) electron-like (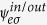) and hole-like (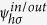) modes having spin projection *σħ*/2, with *σ* = ±1.

Fixing the wave vector modulus 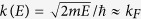, and changing the integration variables, i.e. 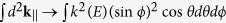, the differential conductance can be derived as 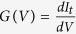 from


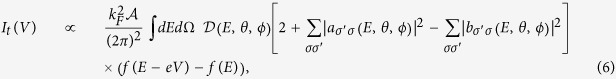


where *f*(*E*) is the Fermi-Dirac distribution, 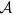 represents the tip cross section, *d*Ω ≡ (sin*ϕ*)^2^cos*θdθdϕ*, while the angular integration is performed over the incidence angles *θ* ∈ [−*π*/2, *π*/2] and *ϕ* ∈ [0, *π*]. The factor 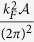 counts the number of transverse modes which participate in the charge transport in the bulk. The finite dimension of the contact (N/F), naturally not included in the asymptotic boundary conditions of the scattering problem, is usually taken into account by introducing the tip shape factor 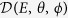 in the current calculation as an exponential^23^ or a Lorentzian^24^ weight. This introduces one more model parameter related to the acceptance tunneling cone. When the spectroscopic features are not significantly affected by the introduction of such weight factor, it is usually neglected in order to simplify the model considering only parameters directly connected to physical measurable quantities. In the BTK formalism, the tip geometry information can be alternatively introduced by limiting the angular integration range (as we did in the calculations) to a maximum incidence angle (rectangular weight factor) defining the tunneling cone. Mathematically, the equivalence of the two procedures is expected for low transparency barriers (high Z values), because in such a case the high angle transmission processes are strongly suppressed^24^.

The normalized differential conductance spectra *G*(*V*)/*G*_*NN*_ (with 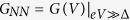) are the physical observables to be compared with the experimental data.

The theoretical model, despite its ballistic nature, is expected to correctly describe physical systems with subdominant diffusive contributions. Indeed, the particle trajectories which contribute to the tunneling current can be classified in two groups: (i) particle trajectories with a scattering angle close to the interface normal direction; (ii) particle trajectories with high incidence angle exploring the transverse dimension of the F layer. The (i)-type trajectories provide the coherent contribution to the tunneling current and retain the quantum mechanical information described by the Bogoliubov-de Gennes formalism (ballistic); (ii)-type trajectories are representative of the diffusive motion along the transverse direction and provide an additive incoherent resistance. This featureless (energy insensitive) spectroscopic background contribution is usually accounted adding a classical resistance to the model, as we did in the calculations. Consequently, the use of a ballistic formalism, corrected by a classical (incoherent) contribution, is suitable for the correct modelling of the physical system. We stress that the BTK theory as well as the several generalized versions of it typically refer to the ballistic description of the phenomenon, verifying the correctness and the applicability of the model by comparison with the experimental results. Moreover, it is widely accepted that ballistic models can be properly used to explain the experimental results obtained in diffusive and/or granular systems, the only significant modification being a larger estimate of the BTK parameter (*Z*) associated to the interface transparency^25^,^26^. Such considerations can be naturally extended to double barrier configurations in which resonant proximity effect is realized.

### Conductance features versus model parameters

Hereafter, we show the conductance features originating from the theoretical model presented before. In discussing the results we use the dimensionless parameter *r* to characterize the ferromagnetic layer thickness *d*, taking the niobium Fermi momentum^27^ 
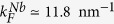
 as the inverse of a reference length scale, i.e. 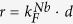. The remaining model parameters are the transparency of the N/F (F/S) interface *Z*_1_ (*Z*_2_) and the (intrinsic) polarization of the ferromagnetic layer *h* = *gμ*_*B*_*M*/*E*_*F*_. The parameter *h* determines the number of spin up (down) electrons 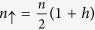 
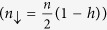
 expressed in terms of the total number of particles *n* = *n*_↑_ + *n*_↓_, so that 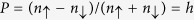.

**Figure 2:**
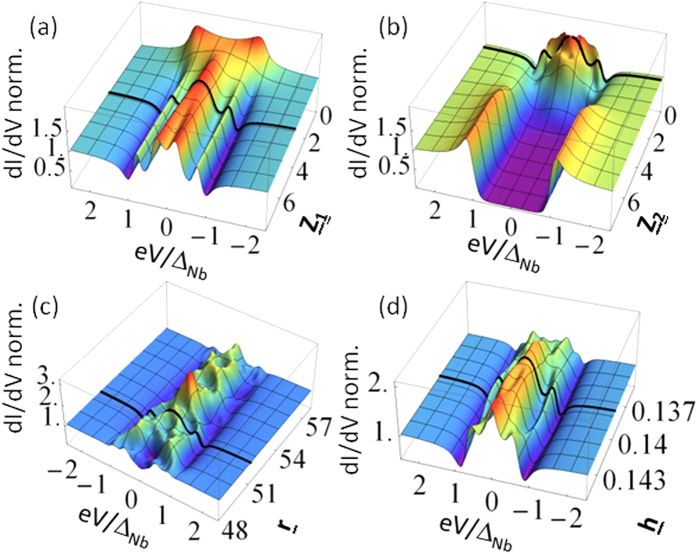
Evolution of the differential conductance spectra calculated using the theoretical model. The black spectrum evidenced in each plot is obtained for *Z*_1_ = 3.85, *Z*_2_ = 0.35, *r* = 51.25, *h* = 0.1403. Different plots are obtained by varying only one parameter (**a**) *Z*_1_, (**b**) *Z*_2_, (**c**) *r*, (**d**) *h* and keeping the other three parameters fixed.

Figure 2 shows the evolution of the conductance spectra as a function of the model parameters (*Z*_1_, *Z*_2_, *r*, *h*). The plots are obtained by keeping fixed three parameters and allowing only one parameter to vary in a range. Thus, we obtain Fig. 2a for 0 < *Z*_1_ < 8, Fig. 2b for 0 < *Z*_2_ < 8, Fig. 2c for 48 < *r* < 58, and Fig. 2d for 0.135 < *h* < 0.145. The solid (black) spectrum, in each panel, corresponds to a reference curve with the model parameters fixed as *Z*_1_ = 3.85, *Z*_2_ = 0.35, *r* = 51.25, *h* = 0.1403.

A completely transparent contact (*Z*_1_ = 0) results in a simple spectrum with two maxima close to the gap energy (see Fig. 2a). Increasing *Z*_1_, there is a slow evolution of the spectra with the appearance of two conductance minima at ±Δ_*Nb*_ and a Zero Bias Conductance Peak (ZBCP) accompanied by two maxima within energy gap. For large *Z*_1_ values, the zero bias peak further splits. Differently, by varying *Z*_2_, the wide variety of conductance features appears only for trasparent F/S barriers (*Z*_2_ < 1), larger *Z*_2_ values causing fully gapped spectra. Thus, the transparent regime is a necessary condition to observe the discussed features. We also notice that in the range *Z*_2_ < 1 the conductance spectra show a moderate dependence on *Z*_2_ (Fig. 2b) with respect to the spectra evolution originated by the remaining parameters (Fig. 2a,c,d). Moreover, the N-F/S configuration favors the formation of bound states in the F-layer^28^ that appear in the differential conductance spectra as subgap resonances. The presence of such states is confirmed by an oscillating behaviour of the ZBC as a function of the *r*-parameter, as observed in Fig. 2c. Indeed, since the emergence of these states is explained in terms of a width-dependent resonance condition, a ZBC modulation is expected by varying the confining dimension (i.e., F-layer thickness parametrized by *r*).

**Figure 3:**
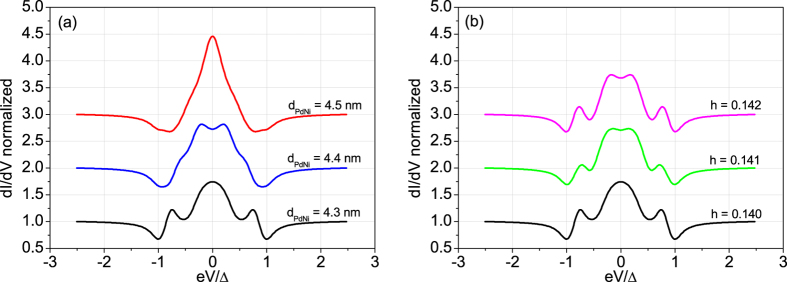
Comparison of conductance spectra calculated for small variations of the parameter (**a**) *r* and (**b**) *h*. The lower (black) spectrum in both panels is the reference conductance spectrum of Fig. 2. In (**a**) *d*_*PdNi*_ corresponds to *r* values according to 
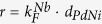
 with 
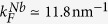
.

From Fig. 2c we notice a strong dependence of the conductance features on the parameter *r*, which can be used for the measurement of the F-layer thickness. The range 48 < *r* < 58 corresponds to a thickness variation of the ferromagnetic layer of less than 1 nm. As an example, in Fig. 3a we show the different spectra obtained for *r* = 51.25 (i.e. 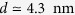), *r* = 52.25 (i.e. 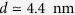) and *r* = 53.25 (i.e. 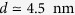). To complete the analysis of the conductance spectra dependence on the various parameters, we show in Fig. 2d the result obtained by varying *h* in the range 0.135 < *h* < 0.145. This parameter gives direct information about the spin polarization *P*. The fast modification of the spectra for small variations of the parameter allows precise estimation of *P*. According to Fig. 3b, the differential conductance features are sensitive to *h* variations of the order of 10^−3^.

### Point contact spectroscopy

We apply the model to the results obtained in a point contact experiment where we realized a N-F/S device by pushing a gold tip on a PdNi(4 nm)/Nb(40 nm) bilayer. The experimental details concerning the setup and sample preparation and characterization are given in the Methods section.

**Figure 4:**
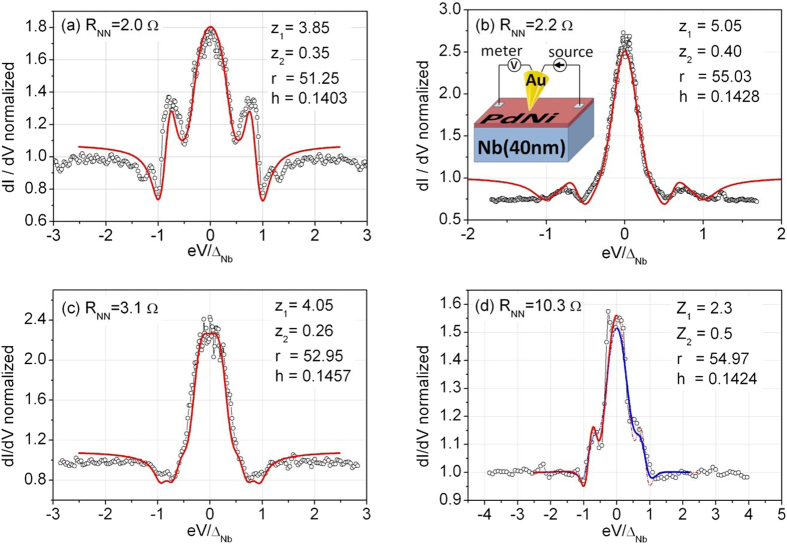
Differential conductance spectra, measured on different contacts at T = 4.2 K. Spectra are classified by *R*_*NN*_, i.e. the high bias resistance. Inset in (**b**) shows a scheme of the setup. Data are normalized to *G*_*NN*_ = 1 (where *G*_*NN*_ = 1/*R*_*NN*_) and compared to curves (solid lines) resulting from the theoretical model. Parameters used to reproduce the data are listed in each plot. All fits are performed by considering a temperature value *T*_*eff*_ = 0.7 K. (**d**) The asymmetry of the spectrum is reproduced by simply assuming a higher temperature for the positive energy side *T*_*eff*_ = 1.1 K.

In Fig. 4, we show a variety of normalized conductance spectra, obtained at *T* = 4.2 K. In some cases, the spectra are characterized by a ZBCP higher than 2 (i.e., *G*(*V* = 0)/*G*_*NN*_ > 2, where *G*(*V* = 0) is the conductance at zero bias), i.e. exceeding the maximum zero bias conductance value (*G*(*V* = 0)/*G*_*NN*_ = 2) expected for a transparent barrier (*Z* = 0) in the standard BTK formulation. Moreover, double conductance dips as well as subgap maxima, with different intensity, are also observed.

To quantitatively analyze the conductance curves reported in Fig. 4a–d, experimental data are compared to theoretically calculated spectra. The fitting parameters are: the N/F barrier strength *Z*_1_, the F/S barrier strength *Z*_2_, the thickness parameter *r* and the spin polarization *h*; superconducting energy gap and the effective temperature being fixed at the values Δ_*Nb*_(0) ≈ 1.24 meV (corresponding to the bulk value expected for a reduced *T*_*c*_ ≈ 7.35 K, see Methods for details) and *T*_*eff*_ = 0.7 K (lower than the bath temperature of 4.2 K).

We notice that some reported conductance features appear too sharp compared to the expected thermal smearing, and require an effective temperature *T*_*eff*_ lower than the bath temperature. Such phenomenon has been already observed for Cu/Nb contacts^7^ and it has been ascribed to possible non-equilibrium transport processes in presence of proximity effect at the interface. Also considering the influence of cooling effect as reported^29^ in N-F/S configuration, the cooling power generally acting on these systems seems to be not enough to cause a temperature reduction of few Kelvin, suggesting that further physical effects should contribute to determine such experimental observation. Alternatively, it has also been predicted that *extraordinary* temperature dependence of the resonant Andreev reflection peak is expected in N/quantum-dot/S systems^30^, the tunneling mediated by discrete energy levels being responsible for an anomalous broadening of the conductance peak with respect the thermal one. Experimentally, such behavior has been reported for N/semiconductor/S systems^31^ and in N/High-Tc-Superconductors constrictions^32^, where the discrete levels could be due to the existence of surface (Andreev) bound states. However, a satisfactory explanation of such effective temperature is still lacking.

All conductance spectra are characterized by a large *Z*_1_ value (2.3 < *Z*_1_ < 5.1) indicating a low transparency of the contact between the gold tip and the PdNi layer; at the same time, significantly lower *Z*_2_ values are always found (0.26 < *Z*_2_ < 0.50) as expected for *in-situ* fabricated PdNi/Nb interface. The extracted *Z*_2_ values confirm that in order to observe these conductance features a transparent F/S barrier is necessary. According to Fig. 4, we can notice that reported spectra are reproduced by 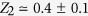. The lowest value (*Z*_2_ = 0.26, Fig. 4c) can be explained either in terms of the slow dependence of the conductance spectra on *Z*_2_ or considering a local variation of the barrier properties (interface roughness, defects, etc.) due to the fabrication process. More interestingly, the remaining parameters involved in the fitting procedure take values in narrow intervals, 51.2 < *r* < 55.1 and 0.140 < *h* < 0.146, from which it is possible to give an estimation of the ferromagnet thickness 4.3 nm < *d* < 4.6 nm in the various sample positions and of the corresponding spin polarization 14.0% < *P* < 14.6%. This finding is compatible with polarization values already reported in literature^33^.

**Figure 5:**
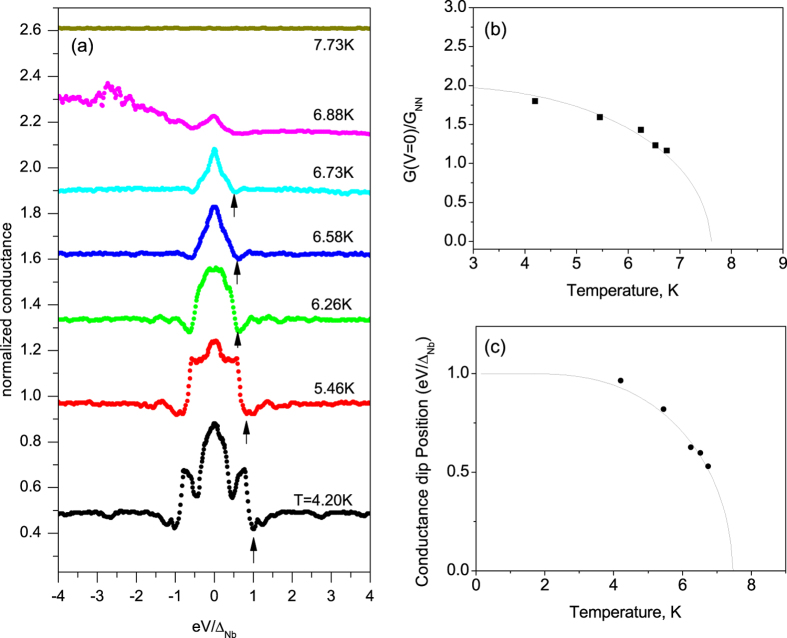
Temperature dependence of the conductance spectrum of Fig. 4a. (**a**) Curves have been shifted for clarity. Black arrows identify the position of the conductance dip at the gap edge. (**b**) Temperature evolution of the relative amplitude of the zero bias conductance *G*(*V* = 0)/*G*_*NN*_ is compared with the theoretical BCS behavior of Δ(*T*). (**c**) Temperature evolution of the conductance dip position. Solid line representing Δ(*T*)/Δ(0) is reported for comparison.

In order to verify that conductance features are strictly related to the superconductivity of niobium, we performed complete temperature dependence of the conductance spectra. We show in Fig. 5 that for *T* = 7.7 K the device is not anymore superconducting and all conductance features are washed out. Moreover, the conductance dip position (black arrows in Fig. 5a) and the amplitude of the zero bias peak both correctly follow the expected BCS behaviour for 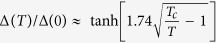, see Fig. 5b,c.

It’s worth mentioning that we used the minimal number of parameters to describe the physical system, where the existence of two interfaces (N/F and F/S parametrized by Z_1_ and Z_2_, respectively) and the ferromagnetic properties of the F-layer (thickness and polarization parametrized respectively by *r* and *h*) are the quantities governing the device physics. Despite the number of parameters, the high sensitivity of the conductance features on the parameter evolution excludes the possibility of degenerate fittings as it has been widely verified during data analysis.

## Discussion

In summary, using the Bogoliubov-de Gennes formalism, we studied the transport properties of N-F/S device in which thin ferromagnetic layer (of the order of *ξ*_*F*_) is deposited on superconducting electrode, realizing a double-barrier structure. The spectroscopic features (i.e., differential conductance spectra) calculated within the theoretical model show a sensitive dependence on the ferromagnet properties (thickness and polarization). This peculiar behavior, originated by the resonant proximity effect, suggests the possibility to use Resonant Andreev Spectroscopy on F/S bilayer as a powerful characterization method to precisely probe local ferromagnetic properties. As a preliminary test of the theoretical expectations, we realized point contact Andreev reflection spectroscopy experiment by pushing a metallic tip on PdNi/Nb bilayer. Differential conductance spectra for several contacts have been measured at low temperature, showing a variety of features (ZBCP, conductance dips at the gap edge, and subgap structures) not expected in single-barrier PCAR theories. Theoretical fittings allowed to consistently explain all measurements: ferromagnet quantities, namely the thickness and the polarization, have been estimated in accordance with the characterization measurements and the relevant literature.

## Methods

The F/S bilayers were grown *in-situ* by a three-target ultra-high vacuum dc magnetron sputtering on Al_2_O_3_ substrates (5 mm × 5 mm) in Argon pressure (few *μ*bar) depositing first a 40 nm thick Nb layer and then a 4 nm thick Pd_0.84_Ni_0.16_ layer. Resistive transition measurements showed that the critical temperature of the bilayer was about 7.2 K compared to 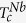 = 8.2 K of a reference (40 nm thick) Nb film. Magnetic and transport characterization of the bilayers has been widely addressed in previous works^34^,^35^,^36^ estimating *ξ*_*F*_ = 3–4 nm^35^,^37^, i.e. the length scale over which the oscillation^37^,^38^,^39^,^40^,^41^,^42^ of the superconducting order parameter is expected in the F-side of the S/F bilayer. The thickness of the ferromagnetic layer covering the superconductor has been chosen to meet the condition *d* ≈ *ξ*_*F*_, in order to favor the coherent interplay between magnetic and superconducting correlations.

PCAR experiments have been performed by pushing a mechanically etched gold tip on the ferromagnetic side of the PdNi/Nb bilayer. The tip is installed on a screw driven chariot to gently approach the sample. The inset is introduced in a liquid helium cryostat to measure current-voltage (I-V) characteristics in the temperature range between 4.2 K and 10 K by conventional four-probe technique. Differential conductance spectra are obtained by numerical derivative. By varying the position and the pressure of the tip on the sample we obtained contact resistances in the range 2 Ω–10 Ω. The transport regime through the contact can be easily estimated by using Wexler’s formula^43^ *R* = 4*ρl*/(3*πa*^2^) + *ρ*/(2*a*) in which the first term gives the Sharvin resistance^44^ describing the ballistic regime and the second one is the Maxwell^45^ resistance describing the diffusive regime. The dominating term will depend on the contact dimension *a*, the resistivity *ρ* of the sample and the mean free path *l* of the charge carriers. For *ρ* = 13 *μ*Ω*cm* (as resulting by direct measurements) and considering that *ρl* = 3.72 × 10^−6^ *μ*Ω*cm*^2^ for niobium^46^,^47^, it comes out that the minimum contact dimension is *a* ≃ 8 nm. The ratio *l*/*a* < 1 gives indication for diffusive contact. However, the extension of BTK theory to diffusive contact has been proven^25^,^26^,^48^ to correctly identify the effect of *P* on the conductance spectra.

The observation of conductance dips has been often ascribed to the formation of thermal contacts. On the other hand, they have also been reported in presence of F/S and N/S point contacts experiments in which the thermal heating is excluded^5^,^49^,^50^,^51^. We do exclude the thermal regime due to experimental evidences in the data. In fact, in the thermal regime, a reduced gap energy is expected^52^ with respect the bulk value, while no evidence of gap reduction is found. Moreover, as reported in ref. 52, the presence of heating effects implies a rising spectroscopic background already at 2Δ_*Nb*_ while we report a flat background well visible up to four times the gap energy. A further experimental observation against the possible formation of a thermal contact is given in Fig. 3 where temperature dependence of conductance spectra is reported. According to D. Daghero *et al*.^53^ the presence of temperature independent high energy tails of the conductance spectra as well as the Andreev signal disappearing at the same critical temperature measured for the bulk system by resistivity characterization demonstrate that the contact resistance has no contribution due to the Maxwell term.

## Additional Information

**How to cite this article**: Romeo, F. *et al.* Resonant Andreev Spectroscopy in normal-Metal/thin-Ferromagnet/Superconductor Device: Theory and Application. *Sci. Rep.* **5**, 17544; doi: 10.1038/srep17544 (2015).
